# Computed Tomography Spectrum of Complications in Usual Interstitial Pneumonia Pattern in a Tertiary Care Hospital: A Descriptive Crosssectional Study

**DOI:** 10.31729/jnma.8706

**Published:** 2024-08-31

**Authors:** Uma Debi, Shritik Devkota, Shayeri Roy Choudhary, Sathya Sagar, Tanka Karki, Mandeep Garg, Nidhi Prabhakar, Sahajal Dhooria, Navneet Singh, Amanjit Bal

**Affiliations:** 1 Department of Radiodiagnosis and Imaging, Postgraduate Institute of Medical Education & Research, Chandigarh, India; 2 Department of Pulmonary Medicine, Postgraduate Institute of Medical Education & Research, Chandigarh, India; 3 Department of Histopathology, Postgraduate Institute of Medical Education & Research, Chandigarh, India

**Keywords:** *complications*, *computed tomography*, *idiopathic pulmonary fibrosis*, *lung cancer*, *usual interstitial pneumonia*

## Abstract

**Introduction:**

Idiopathic pulmonary fibrosis is the most prevalent form of interstitial lung disease, which presents as usual interstitial pneumonia on histopathology and imaging. It leads to significant lung scarring, damage, and fibrosis and is associated with a high degree of mortality, repeated hospital admissions, and oxygen dependence. Many complications are associated with idiopathic pulmonary fibrosis, which further increases the morbidity of patients. High-resolution computed tomography chest is the imaging modality of choice for usual interstitial pneumonia tracking its progression, evaluating treatment response, and detecting potential complications.

**Methods:**

This descriptive cross-sectional study was approved by the Institutional Ethics Committee (Reference number: IEC-INT/2023/Study-1256). Departmental computed tomography report database from November 2017 to June 2018 was reviewed and scans with imaging features consistent with the 'usual interstitial pneumonia' pattern were identified. Total sampling method was used and two independent radiologists, blinded to the patient's clinical information, reviewed the highresolution computed tomography chest scans to assess for imaging features of usual interstitial pneumonia and associated complications.

**Results:**

There were 65 patients reported as unusual interstitial pneumonia pattern. Emphysema and pneumothorax were identified in 4 (6.15%) and 1 (1.53%) scans, respectively. Two (3.08%) scans showed features of pulmonary arterial hypertension. Ten (15.38%) scans exhibited findings consistent with co-existent or superimposed pulmonary infection. Additionally, features of lung malignancy were identified in high-resolution computed tomography scans of 5 (7.69%) patients.

**Conclusions:**

This study sheds light on imaging manifestations of usual interstitial pneumonia complications, aiding radiologists and pulmonologists in earlier diagnosis and improved patient management.

## INTRODUCTION

Idiopathic pulmonary fibrosis (IPF) is the most common fibrosing lung disease, which presents histopathologically and radiologically as usual interstitial pneumonia (UIP).^1,2^ It primarily affects the elderly population and is associated with a high degree of mortality, repeated hospital admissions, and oxygen dependence. Imaging characteristics of the UIP pattern are typical, demonstrating subpleural and basal predominant honeycombing, reticulations, and tractional bronchiectasis.^1^-^3^

Complications such as recurrent pulmonary infections, acute exacerbations, pneumothorax and pneumomediastinum, pulmonary arterial hypertension (PAH), and lung malignancy occurring along the course of UIP increase the disease burden and morbidity.^4-7^ High-resolution computed tomography (HRCT) chest scan is the gold-standard imaging modality for monitoring disease progression, treatment response, and detecting complications in patients with UIP patterns.^5^ This study hopes to address the lack of region-specific data on UIP complications in India.

The aim of this study was to evaluate the complications among patients with UIP patterns on HRCT chest in a tertiary care hospital.

## METHODS

This study employed a descriptive cross-sectional design with retrospective data collection. It was conducted within the Department of Radiology, Postgraduate Institute of Medical Education & Research, Chandigarh, India (a high-volume tertiary healthcare institution). The study received approval from the Institutional Ethics Committee (Reference number: IEC-INT/2023/Study-1256).

To identify participants, we conducted a comprehensive search of the departmental CT report database. We utilized a combination of keywords including "interstitial lung disease (ILD)", "ILD", "usual interstitial pneumonia", "UIP", "idiopathic pulmonary fibrosis", "IPF" or variations like "ILD pattern" or "UIP pattern." This search spanned a period from November 2017 to June 2018. Patients whose computed tomography (CT) reports contained any of these keywords were flagged for further evaluation.

Following the initial search, the CT scans were evaluated for the presence of imaging features consistent with the UIP pattern according to the official guidelines established by the American Thoracic Society, European Respiratory Society, Japanese Respiratory Society, and Latin American Thoracic Society.^3^ Key features considered indicative of a UIP pattern included subpleural and basally predominanthoneycombing,with or without evidence of peripheral traction bronchiectasis or bronchiectasis. Scans without these characteristic findings were excluded from the study.

Additionally, patients with a documented medical history of pulmonary infection, cardiovascular comorbidity, or malignancy before their UIP diagnosis were excluded from the study. This ensured the study focused specifically on patients with UIP and minimized the influence of co-existing conditions on the results.

A total sampling method was employed for patient selection. All the samples within our criteria during the study period were included in this study.

The CT chest scans were performed on the multidetector CT machines set up in the department; the third generation dual-source CT 128 slice scanner: Somatom Definition Flash (Siemens Healthcare, Forchheim, Germany) or CT 256 Slice scanner (Philips Brilliance iCT). Two experienced radiologists independently reviewed the chest CT scans of 65 consecutive patients. They were blinded to the patients' clinical information and focused on identifying imaging patterns consistent with UIP and any potential complications. Associated pathologies and complications such as emphysema, pneumothorax and pneumomediastinum, pulmonary infection, PAH, and lung malignancy were reported. Any discrepancies in interpretation between the radiologists were resolved through mutual discussion and consensus.

For pulmonary infection or pneumonia, radiological findings such as centrilobular nodules with a tree-in-bud pattern, segmental or lobar pattern, areas of central cavitation, or pleural empyema were included.^8^ Additionally, the presence of mediastinal lymph nodes was also supportive of infective pathology. Pulmonary infection was further confirmed by bronchoalveolar lavage workup and image-guided sampling if needed.

Lung malignancy was suspected based on the presence of masses with surrounding distorted lung architecture or spiculated margins on HRCT scans. These findings were subsequently confirmed through histological examination.^9^ Based on specific radiological measurements obtained from the HRCT scans, PAH was suspected. These included a ratio of the main pulmonary artery (MPA) diameter to the aorta diameter greater than 1, and an MPA diameter exceeding 29 mm (with a slightly lower threshold of 27 mm for females).^10^

Data collected from the imaging evaluation was entered, compiled, and analyzed using Microsoft Excel software. Continuous data, such as measurements, were presented as averages with standard deviations. Categorical data, like the presence or absence of specific conditions, were presented as counts and percentages.

## RESULTS

There were 65 patients with chest CT characteristic of UIP pattern. The average age of patients was 59 years with minimum age of 24 and maximum of 91 years. There were 43 (66.15%) male in the study population.

Complications in UIP was observed in 23 (35.38%) patients. Co-existing or superimposed pulmonary infection 10 (15.38%) was one of the CT spectrum of complications observed in those UIP (Table 1).

One (1.54%) additional patient presented with confirmed metastasis from an abdominal malignancy. Among the 5 (7.69%) cases with lung masses, 4 (6.15%) tumors were present in the upper lobes, 1 (1.53%) lesion was subpleural based in the left lower lobe, and 1 (1.53%) was a large infiltrative central mediastinal mass. Architectural distortion was a feature seen with lung masses. All cases of lung malignancy exhibited enlarged lymph nodes near the lungs and in the mediastinum. Liver metastases were identified in one patient with a lung mass.

**Table 1:** HRCT spectrum of complications of usual interstitial pneumonia pattern (n = 65).

CT Spectrum of Complications of UIP Pattern	n (%)
Co-existent or superimposed pulmonary infection	10 (15.38)
Lung malignancy	5 (7.69)
Emphysema	4(6.15)
Pulmonary arterial hypertension	2 (3.08)
Pneumothorax	1 (1.53)
Abdominal malignancy	1 (1.53)

## DISCUSSION

The pattern for UIP on imaging is subpleural and basal predominant fibrosis, honeycombing with or without peripheral tractional bronchiectasis or bronchiectasis.^2,3^ In our study, we have analyzed the spectrum of the possible complications that are associated with this disease.

Emphysema is a common finding in HRCT of patients with IPF, seen in anywhere between 8-50.9% of patients.^11^ We found emphysema in 6.15% of the scans. In our study, 3.08% of the patients had CT features of PAH when we measured the MPA and aorta at the pulmonary bifurcation. Computed tomography can be considered a handy non-invasive screening procedure for PAH in patients, and it is also used in IPF patients, however, it is less reliable in IPF due to pulmonary fibrosis which causes mediastinal traction and dilatation of the arteries. None of the non-invasive screening modalities independently or in combination are very effective in screening PAH in IPF.^10,12^ Pneumothorax was seen in 1.53% of our study subjects. However, the incidence of pneumothorax in patients with IPF is reported to be between 3-11%.^13^

One of the most common complications and a frequent cause of admission of IPF patients is pulmonary infection. In a cohort study, it was second to only acute exacerbation as a cause of hospital admission.^6^ In our study, we observed ten CT scans with definitive features of lung infection; the most common pattern being centrilobular nodules, tree-in-bud opacities, and small sub-segmental consolidations.^8^ A small loculated empyema was seen in one patient.

The most common infections in these patients are from Mycobacterium species and fungal infections by Aspergillus. The occurrence of aspergillosis with fungal ball formation has been described to co-exist with a variety of pathologies, commonly in pre-existing lung cavities like tuberculosis.^5,6^ The presence of irregular soft tissue in a cavity with an air crescent sign and the presence of motion of this soft tissue on a prone HRCT scan is virtually diagnostic of aspergilloma.^6^ In our study, we did identify one patient with the presence of an irregular cavity with rounded hypodense soft tissue within the cavity to suggest the possibility of aspergilloma.

Patients with UIP-IPF have a well-established association with primary lung carcinoma.^9,14,15^ The incidence of cancer is much higher in this subset of the population due to increased parenchymal damage and scarring with repeated inflammatory insults.^14^ Carcinoma in association with UIP is more common in males, in older age groups (common over 60 years), and with a history of smoking.^14^ In our study, we found lung cancer in 5 (7.69%) patients. We identified four tumors in the upper lobes, contrary to published data.^14^ One lesion was subpleural based in the left lower lobe, and another was a large infiltrative central mediastinal mass lesion. A large lesion with architectural distortion was the most common finding. Associated hilar and mediastinal lymphadenopathy was seen in all cases.

Squamous cell carcinoma and adenocarcinoma are the most common types of lung cancer found in patients with IPF, with adenocarcinoma being slightly less frequent than squamous cell carcinoma.^9^ These tumors tend to be larger and situated on the periphery, particularly in the lower lobes, often developing within areas of extensive fibrosis characteristic of IPF. They can appear as either single, lobed masses or large cavitated lesions.^9^ Adenocarcinomas with a bronchoalveolar pattern may exhibit ground-glass opacities and soft tissue masses around the bronchovascular structures, demonstrating the characteristic "lepidic growth" pattern, although this is best confirmed through microscopic examination. The presence of multiple tumors or lesions occurring at the same time (synchronous lesions) is also common.^9,14,15^

A significant challenge in diagnosing lung cancer in patients with IPF is the extensive architectural distortion caused by the underlying fibrosis. This distortion can make it difficult to distinguish between cancerous and fibrotic tissue, especially in cases with fibrosis that appear mass-like.^9^ Careful comparison of serial CT scans looking for subtle changes in size, character, or the development of new areas of opacity within the lung tissue can raise suspicion for an underlying malignancy. Additionally, lymphovascular invasion and involvement of the lymph nodes in the mediastinum are relatively common findings in lung cancer associated with IPF.^9^

The overall prognosis for patients with both lung cancer and IPF is demonstrably worse compared to those with lung cancer alone. This increased mortality risk is attributed to the greater frequency of acute exacerbations and other complications associated with ILD.^14^ The presence of ILD itself is a major negative prognostic factor for lung cancer patients. Studies have shown that individuals with lung cancer who lack co-existing IPF have a significantly longer survival period and experience a more favorable clinical course compared to those with both diagnoses.^15^ Treatment options for lung cancer in IPF patients include chemotherapy and surgical resection, although the specific management strategies fall outside the scope of this discussion.

Acute exacerbation of ILD represents episodes of sudden worsening in respiratory function and clinical deterioration in patients with ILD. While the proposed causes of AE-ILD have been extensively described in the literature, the exact mechanisms by which these events occur remain largely unknown.^16^ AE-ILD is diagnosed through a combination of clinical features (duration less than one month) with specific chest CT findings.^17^ Ruling out other causes (myocardial infarction, pulmonary embolism, cardiogenic edema, infections) remains crucial.^4^

We acknowledge certain limitations in our study. The cross-sectional design limits establishing causality between UIP and complications. We analyzed only chest CT scans, excluding clinical data and long-term follow-up. The confirmation of PAH relied solely on imaging, lacking invasive pressure measurements. Our study design and inclusion criteria limited the evaluation to patients with a UIP pattern on HRCT scans. This precluded analysis of patients with histopathological confirmation of ILD lacking imaging features of UIP pattern. Furthermore, the potential confounding effect of smoking and other established risk factors for emphysema was not addressed. Finally, the single-center design and small sample size may limit generalizability. Despite these limitations, our study offers a preliminary analysis of complications associated with UIP using HRCT chest scans.

## CONCLUSIONS

Patients with UIP often experience severe lung scarring, and frequent complications, and require regular chest CT scans to monitor disease progression and identify potential complications. This study investigated the radiological spectrum of such complications in UIP patients, offering valuable insights for clinicians and radiologists. Future large-scale, multicenter studies with long-term follow-up are warranted to further elucidate the relationship between UIP and these complications.
